# Genetic aspects of pituitary carcinoma: A systematic review

**DOI:** 10.1097/MD.0000000000005268

**Published:** 2016-11-28

**Authors:** Zijiang Yang, Ting Zhang, Heng Gao

**Affiliations:** a Department of Neurosurgery, Jiangyin People's Hospital Affiliated to Nantong University; b Central Laboratory, Jiangyin People's Hospital Affiliated to Nantong University; c Department of Neurosurgery, Jiangyin people's Hospital Affiliated to Nantong University, Jiangyin, Wuxi, China.

**Keywords:** gene expression, pituitary carcinoma, potential clinical application value, protein expression, systematic review

## Abstract

**Background:**

Pituitary carcinoma (PC) is a rare type of malignant intracranial neoplasm defined as distant metastasis of pituitary adenoma (PA). Although PC incidence is low because only 0.1% to 0.2% of PAs ultimately develop into PCs, the prognosis is poor and 66% of patients die within the first year. Existing therapeutic measures, including surgical removal, chemotherapy, and radiotherapy, have limited effectiveness. The lack of efficacy of current treatments is largely caused by the limited understanding of the molecular pathogenesis of PA and the malignant transformation to PC. Therefore, the aim of this systematic review was to summarize published research regarding gene and protein expression in PC to clarify the molecular mechanisms underlying PC genesis and development and identify new candidate diagnostic biomarkers and therapeutic targets for potential use in personalized treatment of PC.

**Methods:**

We followed the PRISMA guidelines to plan and conduct this systematic review. PubMed, Embase, and Web of Science databases were searched for relevant studies conducted before December 16, 2015 describing the association of PC with gene expression at the mRNA and protein levels. MeSH terms combined with free terms were used to retrieve the references.

**Results:**

In total, 207 records were obtained by primary search, and 32 were included in the systematic review. Compared with normal pituitary gland and/or PA, 30 and 18 genes were found to have higher or lower expression, respectively, in PCs using different analytical methods. Among them, we selected 9 upregulated and 7 downregulated genes for further analysis based on their identification as candidate treatment targets in other cancers, potential clinical application, or further research value.

**Conclusion:**

Previous studies demonstrated that many genes promote PC malignant transformation, angiogenesis, invasion, metastasis, and recurrence. Although most of these genes and proteins have not been fully analyzed with regard to their downstream mechanisms or potential diagnostic and therapeutic application, they have the potential to become candidate PC biomarkers and/or molecular targets for guiding personalized treatment. Modern advanced technologies should be utilized in future research to identify more candidate genes for PC pathogenesis, as precisely targeted gene therapies against PC are urgently required.

## 1 Introduction

Pituitary carcinoma (PC) is a malignant tumor occurring in the pituitary gland, located at the base of the brain. The characteristic feature of these tumors is metastatic spread into other areas of the brain and spinal cord as well as outside the central nervous system. Although PCs are rare, constituting only 0.1% to 0.2% of pituitary adenomas (PAs), the prognosis is extremely poor and 66% of patients die within the first year. Most PCs exhibit endocrine function, mitotic activity, and aggressive behavior. In total, 88% of PCs have endocrine activity; among them, 42% secrete adrenocorticotropic hormone (ATCH), 33% secrete prolactin (PRL), 6% secrete gonadotropic hormone (GH), 5% secrete luteinizing hormone and follicle-stimulating hormone, and 1% secrete thyroid-stimulating hormone.^[1]^ The latency between the first presentation of PA and its transformation to PC depends on the type of endocrine activity; thus, the average latency period for ACTH-secreting PCs is 9.5 years, and that of PRL-secreting PCs is 4.7 years. Most PCs are characterized by systemic rather than encephalomyelic metastases: 71% and 57% of ACTH- and PRL-secreting PCs, respectively, show systemic dissemination.^[2]^ The invasion mechanism of PC is similar to that of other tumors and is based on the ability to freely pass through the extracellular matrix (ECM) and vascular endothelium and spread by blood circulation, and accumulating evidence suggests a role for deregulation of gene expression.^[3]^ However, the mechanisms underlying the metastatic activity of PCs are still unclear because the variation of gene expression in primary PCs is not always consistent with pituitary metastases.^[4]^

Existing therapeutic measures, including surgical removal, chemotherapy, hormone-targeting drugs, molecular therapy, and radiotherapy, are not always helpful. The current treatment of choice for PC is transsellar exeresis combined with radiotherapy. However, traditional radiation does not provide a survival advantage.^[5]^ Modern gamma-knife radiosurgery has been shown to be effective in controlling PC growth over 3 years,^[6]^ whereas peptide receptor radiotherapy with ^117^Lu DOTATATE was shown to stop PC growth for more than 4 years.^[7]^ Still, the benefit of these modern radiotherapeutic approaches needs further validation. The therapeutic effects of chemotherapy are controversial. The most efficient agent is temozolomide (TMZ), which improves outcomes for PCs characterized by methylation of the *O*6-alkylguanine DNA alkyltransferase-encoding gene (*MGMT*)^[8]^ and/or upregulation of a mismatch repair protein (MSH6).^[9]^ Application of cyclo-hexyl-chloroethyl-nitrosourea (CCNU) combined with 5-fluorouracil (5-FU) has demonstrated efficacy in the treatment of different PC subtypes,^[10]^ whereas continuous administration of the anti-VEGF antibody bevacizumab was shown to inhibit angiogenesis and promote fibrosis, which was correlated with suppression of PC growth.^[11]^ Thus, understanding the genetic pathogenesis of PC is essential for the accurate and effective treatment of the disease.

The identification of PC-specific molecular biomarkers would contribute to the improvement of current diagnostic criteria and to the development of targeted therapeutic approaches, which would potentially increase the long-term survival of PC patients. In this systematic review, we aimed to analyze the results of previous studies regarding differential expression of genes in PCs to identify genetic mechanisms associated with malignant transformation and progression of PC.

## 2 Methods

### 2.1 Search strategy

We conducted this systematic review following the PRISMA guidelines to identify all published studies on gene expression changes in PCs compared with normal pituitary glands and/or PAs. The strategy included a search of PubMed, Embase, and Web of Science databases to retrieve relevant studies published before December 16, 2015 using MeSH terms combined with free search terms. A combination of the following keywords was used: Pituitary Carcinoma OR Pituitary Carcinomas OR Cancer of the Pituitary OR Pituitary Cancer OR Pituitary Cancers, Gene Expression, Oligonucleotide Array Sequence Analysis, Microarray Analysis, Reverse Transcriptase Polymerase Chain Reaction, Immunohistochemistry (IHC), and Western Blotting. We did not directly use pituitary neoplasm as a MeSH search term because pituitary neoplasms comprise many pituitary tumor types. No other restrictions were imposed, and no attempt was made to obtain unpublished results.

### 2.2 Study selection

The selection of published material was conducted based on initial screening of titles or abstracts followed by screening of full-text reviews. Manuscripts were considered eligible if they met the following criteria: PCs were compared with normal pituitary glands and/or PAs; analyzed tissues were of human origin; gene expression was experimentally evaluated. The exclusion criteria were PC tissues removed after drug treatment; metastasis to pituitary gland; morphological analysis; mentioning PCs without molecular studies; and animal experiments.

### 2.3 Data extraction and quality assessment

The information on the retrieved studies included: the last name of the first author and publication year; differentially expressed genes; experimental approach; and control (tissue type and source). The initial review of the search results was accomplished by 1 author (YZJ). The analyzed tissues, methods, and results of each full-text article were reviewed to satisfy the selection criteria and assess the quality of the text. No studies were excluded as a result of poor quality of methods or unsatisfactory results. The final information was independently extracted by the 2 authors (YZJ and GH). Any discrepancies were resolved by discussion.

### 2.4 Ethical approval

Because the data for this systematic review were collected from published literature, patient consent was not needed in our study. Therefore, we did not ask the ethics committee or institutional review board for approval of the study.

### 2.5 Potential for bias

The initial search of databases was strictly based on the relevance between the primary research and our topic. In addition to the extracted information described above, no other parameters such as authors other than the first, research institutions, region, source, and journal's grade were considered. Preliminary screening was accomplished by 1 author (YZJ) and then rechecked by the other author (GH) according to the defined criteria to reduce errors. The information was extracted from the full text independently by each author. These efforts were directed toward conducting the analysis with minimal bias.

## 3 Results

### 3.1 Literature search

**Figure 1:**
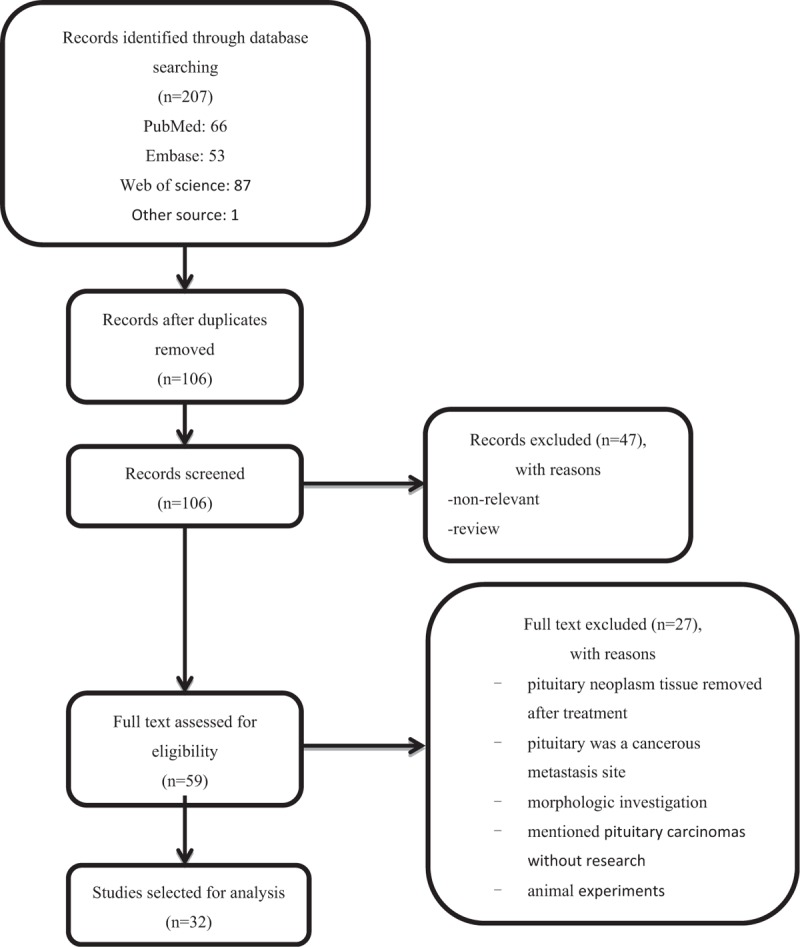
Flow chart of the study selection process.

The PRISMA-based flow chart showing study selection is presented in Fig. 1. In total, 207 initial studies were extracted from PubMed, Embase, and Web of Science databases, and 106 articles remained after removal of duplicate records. In total, 47 reviews and unrelated articles were excluded, and 59 were selected for the next step of analysis. As a result, 32 articles were accepted for systematic review based on the defined inclusion and exclusion criteria.

### 3.2 Study characteristics

The characteristics of the selected studies are presented in Table 1. In total, 48 genes were analyzed and described in journal publications before December 16, 2015; among the selected genes, 30 were upregulated and 18 downregulated in PCs compared with normal pituitary tissues and/or PAs. Most PAs used as the control were classified by invasion phenotype rather than secretory type. Many researchers used multiple methods to detect gene expression. Among these methods, reverse transcription (RT)-PCR was the most common technique (10 articles), followed by in situ hybridization (ISH, 4 articles), gene microarray (3 articles), miRNA microarray (2 articles), northern blotting, capillary gel electrophoresis, DNA sequencing, and Southern blotting (1 article each). Protein expression was most frequently analyzed by IHC (27 articles), which was the only analytical method in 15 studies and was combined with RT-PCR in 7 studies. Western blotting was used in 5 studies, and TRAP/TRAP-HPA (to detect telomerase activity) and the avidin-biotin-peroxidase complex method were each used in 1 study.

**Table 1:**
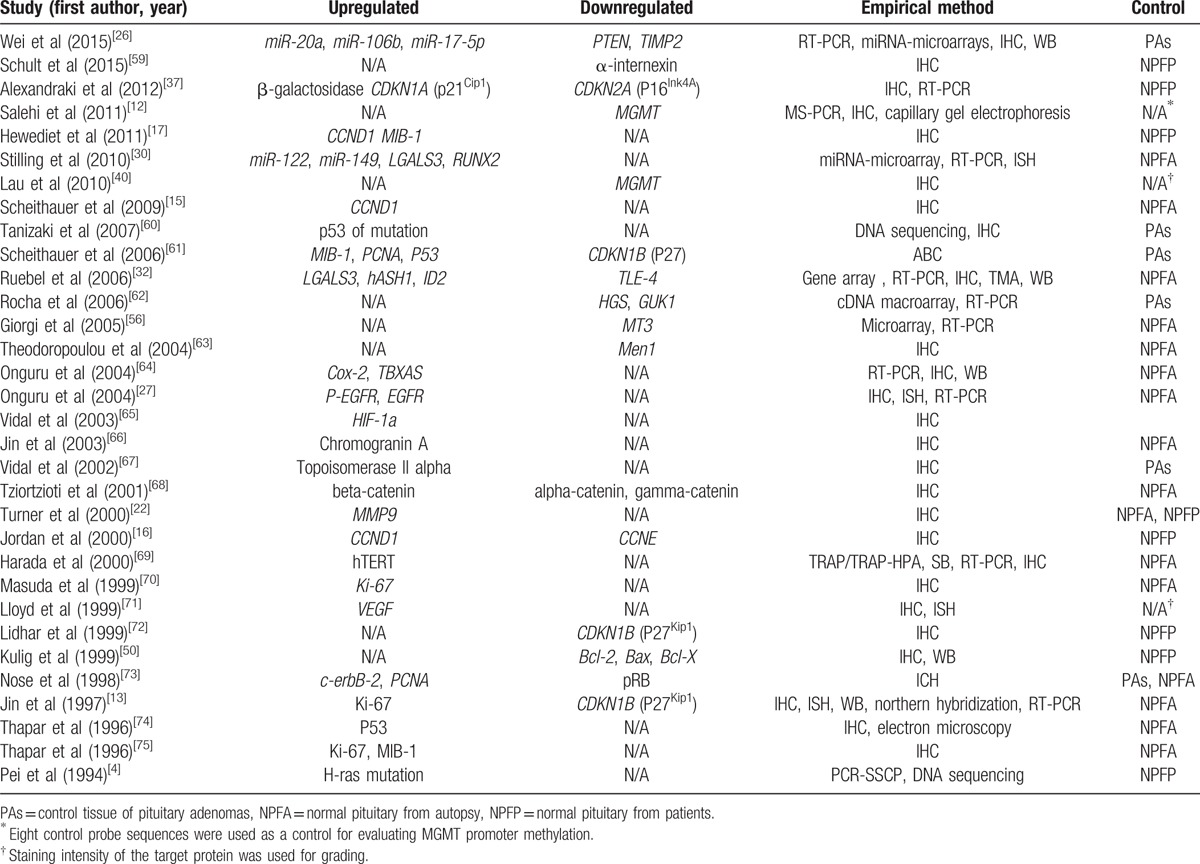
Characteristics of the 32 studies included in this systematic review.

In several studies, gene expression was evaluated by PCR, and the corresponding protein expression was analyzed by IHC; the results were consistent except for those related to MGMT, cyclin-dependent kinase inhibitor 1B (p27^Kip1^), and hepatocyte growth factor-regulated tyrosine kinase substrate (HGS). *MGMT* methylation status was not concordant with MGMT expression,^[12]^ and mRNA levels of *CDKN1B* (p27^Kip1^) and *HGS* were similar to those in normal pituitary glands and PAs, whereas the expression of the corresponding proteins gradually decreased during the progression from normal tissue to malignancy.^[13]^

### 3.3 Main analysis

In the retrieved studies, many genes differentially regulated in PCs were described in the retrieved studies. Further gene selection was based on objective and subjective criteria. The objective criteria were confirmation of involvement in PC development in multiple studies and use as drug targets in the treatment of PC, PA, and/or other tumors. The subjective criteria were the potential for clinical application of early diagnosis and molecular targeted therapies or insufficient functional analysis both in vitro and in vivo. In addition, micro(mi)RNAs as important post-transcriptional regulators were included in the analysis if their expression in microarrays was further validated by RT-PCR. The selected genes are presented in Table 2.

**Table 2:**
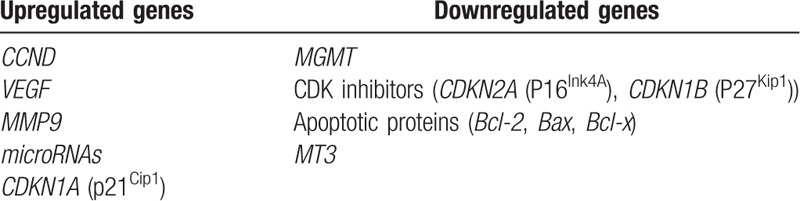
Summary of highlighted genes.

### 3.4 Genes upregulated in PCs

#### 3.4.1 CCND1/cyclin D1

Cyclins are a family of proteins that controls cell cycle progression by activating cyclin-dependent kinases (CDKs), which regulate processes involved in cell division, including microtubule formation and chromatin remodeling, in a phosphorylation-dependent manner. Cyclin D1, encoded by the *CCND1* gene, binds and activates CDK4, which phosphorylates retinoblastoma (pRb) protein and is, in turn, inhibited by p21^Cip1^ and p27^Kip1^, thus regulating the G1-to-S phase transition.^[14]^*CCND1* is overexpressed in a variety of tumors, including PCs. Scheithauer et al^[15]^ and Jordan et al^[16]^ demonstrated that cyclin D1 was upregulated in the majority of PAs compared with normal pituitary glands as well as in invasive/atypical PAs and PCs compared with noninvasive PAs.

Another study showed that cyclin D1 was more highly expressed in pituitary tumors and was associated with cell proliferation, tumor recurrence, and metastatic potential, suggesting that nuclear cyclin D1 expression is a reliable biomarker of aggressive behavior in pituitary tumors.^[17]^ A recent study suggested new functions of cyclin D1, such as stimulation of cell migration and invasion, enhancement of angiogenesis, regulation of transcription factor activation, induction of chromosomal instability, and control of miRNA expression.^[14]^ However, these findings have not been confirmed by further investigations in PCs. Research in these directions can increase the current understanding of PC occurrence and provide a theoretical basis for the development of selective CDK4/6 inhibitors for precise treatment of PC.

#### 3.4.2 VEGF

VEGF expression is strongly associated with tumor angiogenesis, cell proliferation, invasion, and metastasis. VEGF levels were higher in PCs compared to those in PAs, suggesting that VEGF is an important growth factor associated with malignant transformation.^[37]^ Patients with GH- and PRL-secreting PAs treated with octreotide and bromocriptine, respectively, before surgery had a lower level of VEGF than untreated patients.^[37]^ These findings indicated that octreotide and bromocriptine suppress the expression of GH and PRL, and furthermore, we could deduce that overexpression of GH or PRL promotes the expression of VEGF in PCs. The anti-VEGF antibody bevacizumab effectively inhibits PC growth,^[11]^ suggesting that targeting of VEGF signaling may become a new adjuvant treatment. The VEGF receptor (VEGFR) is overexpressed in aggressive PAs and is related to poor outcomes, and may thus also present a potential therapeutic target. VEGFR inhibitors have provided favorable oncologic outcomes in nonsmall cell lung cancer in which chemotherapy or other targeted therapies had failed. However, no studies have been published regarding the use of VEGFR inhibitors for PC treatment.^[18,19]^ The expression of VEGF in tumors is induced by oncogenes such as hypoxia-inducible factor 1^[20]^ and mutant H- and K-Ras GTPases.^[21]^ Matrix metalloproteinase 9 (MMP-9) has been implicated in pituitary tumor behavior^[22]^ and can promote the secretion of VEGF and tumor growth.^[23]^ Other factors involved in tumor vasculogenesis are cyclooxygenase-2 and the downstream thromboxane A synthase 1 (TBXAS); these molecules play an important role in pituitary tumor progression by promoting vascular growth and are also more highly expressed in PCs than in normal pituitary glands.^[27]^ However, although these genes have been shown to be deregulated in PCs, no experimental evidence is available regarding the relationship between their expression and VEGF level in PCs.

#### 3.4.3 Matrix metalloproteinase 9

MMP-9 belongs to the zinc metalloproteinase family of proteins, which are involved in the remodeling and degradation of the ECM and thus facilitate the migration of tumor cells. MMP-9 plays a crucial role in many aspects of cancer progression, such as growth, angiogenesis, tumor cell invasion, motility, and hematogenous, and lymphatic tumor dissemination.^[24,33]^ MMP-9 is significantly upregulated in PCs and recurrent PAs compared to normal pituitary glands and in invasive compared to noninvasive PAs, suggesting that MMP-9 is associated with aggressive behavior and promotes malignant transformation of pituitary tumors. Similarly, it has been found that increased MMP-9 levels and decreased levels of tissue inhibitors of metalloproteinases (TIMP-1 and TIMP-2) stimulate tumor progression and metastasis in esophageal cancer, whereas TIMP-2 also shows decreased levels in PCs.^[25,26]^ Whether the increase in MMP-9 levels and decrease in TIMP-2 levels promote tumorous malignant transformation in PCs requires further research. MMP-9 has been shown to act through activation of EGFR signaling, and both EGF and EGFR were found to be overexpressed in PCs.^[27]^ Another study corroborated the association between the EGF pathway and MMP-9 by demonstrating that EGFR signaling downstream of EGF regulated MMP-9 secretion and promoted the migration and invasion of human ameloblastoma cells.^[28]^ These data suggest the potential of synthetic MMP-9 inhibitors in PC treatment. However, research on the relationship between MMP-9 and EGFR in PC is limited, and the involvement of MMP-9 expression in PC angiogenesis remains unknown. Therefore, further studies are required to elucidate the potential of manipulating MMP-9 in the control of PC development.

#### 3.4.4 MicroRNAs

MiRNAs are short noncoding RNA molecules that regulate gene expression at the post-transcriptional level by selectively targeting complementary mRNA for degradation. Depending on the degree of complementation, miRNA may either fully or partially represses mRNA translation; moreover, it can also activate gene expression by binding to the 5′-untranslated region of the target mRNA.^[29]^ It is established that miRNAs are important multifunctional regulators involved in many intracellular mechanisms that control cell cycle, proliferation, differentiation, apoptosis, invasion, and carcinogenesis. Therefore, we searched for indicators of miRNA involvement in PC development and retrieved 2 relevant articles.

Wei et al^[26]^ found that miR-20a, miR-106b, and miR-17-5p were upregulated in nonfunctioning metastatic PC compared to the primary neoplasm and in metastatic PC and atypical PAs compared to typical PA; the involvement of these miRNAs in tumor progression was attributed to inhibition of phosphatase and tensin homolog deleted on chromosome 10 (PTEN) and TIMP-2. However, the effect of miR-20a, miR-106b, and miR-17-5p in PCs has not been validated by in vitro experiments.

Stilling et al^[30]^ found that miR-122 and miR-493 were significantly overexpressed in ATCH-secreting PCs compared with PAs by using a miRNA microarray. MiR-493 was also found to be upregulated in PAs compared with the normal gland, but there was no difference in miR-122 expression between normal and PA tissues, suggesting that miR-122 is involved in the malignant transformation of pituitary tumors. However, the mechanism modulating the effects of miR-122 on the malignancy of pituitary neoplasms has not been further investigated.

MiR-493 was also shown to be significantly upregulated in ATCH-PCs, and the MiRBase database predicts that miRNA-493 can target *RUNX2* and *LGALS3* mRNA.^[31]^ The *LGALS-3* gene encodes the adhesion protein galectin-3, which is a downstream target of the transcription factor RUNX2 and regulates pituitary tumor cell proliferation and apoptosis. Ruebe et al^[32]^ showed that *LGALS-3* was more highly expressed in PRL- and ACTH-secreting PAs and ACTH-secreting PCs compared with GH-PAs and nonsecreting PAs. As miRNAs can not only inhibit but also induce the translation of targeted mRNAs, it should be examined whether the increase in miR-493 levels stimulates galectin-3 expression in PCs. Overall, the data on the roles of miRNA in PC are limited, and further investigation is necessary to validate the utility of miRNA for PC diagnosis and treatment.

#### 3.4.5 p21^Cip1^

Cyclin-dependent kinase inhibitor 1 (p21^Cip1^) belongs to the WAF/KIP family and is involved in the regulation of the cell cycle. P21^Cip1^ binds to and inhibits the activity of cyclin-CDK2, -CDK1, and -CDK4/6 complexes, promoting cell cycle arrest at the G1 phase.^[33]^

p21^Cip1^ is overexpressed in hormone-secreting PAs, especially in GH-PAs,^[34]^ and no mutations have been observed in the encoding gene.^[35]^ Chesnokova et al^[36]^ reported that p21^Cip1^ suppressed pituitary tumor growth, which was correlated with hormonal secretion type, and hypothesized that p21^Cip1^ overexpression accounted for the very low incidence of PCs. However, p21^Cip1^ was also found to be highly expressed in PCs, although 80% of the analyzed tumors were hormone-secreting.^[37]^ Similar to giant cell tumors, upregulation of p21^Cip1^ appears to promote cyclin D1 accumulation, contributing to tumor formation.^[38]^ Thus, the results regarding the role of p21^Cip1^ in pituitary tumors are conflicting, and it is unclear whether p21^Cip1^ is involved exclusively in hormonal secretion, or also in other mechanisms underlying PC initiation and progression.

### 3.5 Genes downregulated in PCs

#### 3.5.1 O6-methylguanine-DNA methyltransferase

*O*6-methylguanine-DNA methyltransferase (MGMT) is a key enzyme for repairing the mutagenic DNA lesion *O*6-methylguanine caused by environmental alkylating agents. If MGMT expression is low, DNA damage would result in the G/C to A/T transition, oncogene activation, and tumor formation.^[39]^

PCs are characterized by *MGMT* promoter methylation and low levels of enzyme expression. MGMT downregulation was observed in a significant subset of PCs and invasive PAs, independently of the type of secreted hormones.^[40]^ These data were corroborated in another study showing *MGMT* promoter methylation in a significant portion of PCs independently of endocrine activity.^[12]^ Because methylation status was not always concordant with MGMT expression in the same samples, the molecular mechanisms underlying MGMT activity in pituitary tumors remain unknown.

TMZ is an alkylating agent that induces modifications at the N-7 or O-6 positions of guanine residues in DNA; it is cytotoxic and promotes tumor cell apoptosis. Low MGMT immunoreactivity is associated with sensitivity to TMZ treatment in patients with PC and aggressive PA, and MGMT expression has been described as a biomarker to predict tumor response to TMZ in case reports and small case series.^[41]^ However, Preusser et al^[42]^ observed poor correlation between MGMT immunoreactivity and *MGMT* promoter methylation status, and no significant association between MGMT expression and prognosis for glioblastoma multiforme patients. These data conflict with the mechanism linking low MGMT expression in tumors to the increase in DNA alkylation by TMZ, which should result in tumor cell death; therefore, further research is needed to clarify the function of MGMT in pituitary tumors and provide a theoretical basis for clinical use of TMZ in the treatment of PC patients.

#### 3.5.2 CDK inhibitors

P16^Ink4A^ belongs to the INK family of CDK inhibitors and suppresses the activity of CDK4 and CDK6, causing cell cycle arrest.

Downregulation of p16^Ink4A^ has been found in a wide variety of human cancers, including pituitary neoplasms. Decreased p16^Ink4A^ levels are commonly observed in all PAs and inversely correlated with tumor size and recurrence; dependence on PA type has also been observed.^[43,44]^ In the majority of PAs, changes in p16^Ink4A^ expression are not associated with gene mutation or loss, suggesting that genetic transformation of p16^Ink4A^ is not involved in PA development.^[45]^ Simpson et al^[46]^ demonstrated that the pRb/p16/cyclin D1/CDK4 pathway is frequently deregulated in PAs, facilitating tumor formation. Consistent with these results, Alexandraki et al^[37]^ showed that p16^Ink4A^ was significantly downregulated in PAs and PCs. In contrast, Yi et al^[47]^ reported that p16^Ink4A^ levels analyzed by ICH and ISH were decreased in PAs. These studies were confined to evaluation of gene expression levels with no related functional data. Thus, the involvement of p16^Ink4A^ in PC development needs further validation.

P27^Kip1^ belongs to the WAF/KIP family of CDK inhibitors and arrests the cell cycle at the G1 to S transition. The inhibitor preferentially binds CDK2 and blocks the activity of the cyclin E-CDK2 complex, but can also interact with CDK4 and suppress cyclin D-CDK4 activity.^[48]^

Using ICH, it has been revealed that p27^Kip1^ is most highly expressed in normal pituitary glands and is completely absent in PCs,^[13,38]^ suggesting that the loss of p27^Kip1^ expression leads to malignant transformation in PCs. However, no significant differences in mRNA expression of the encoding *CDKN1B* gene were observed among normal pituitaries, PAs, and PCs.^[13]^ Therefore, the specific role of p27^Kip1^ in PCs should be addressed in the future.

#### 3.5.3 Apoptotic proteins of the Bcl-2 family

The Bcl-2 family comprises antiapoptotic proteins such as Bcl-2 and Bcl-X_L_ and pro-apoptotic members such as Bax and Bcl-X_S_.^[49]^

Kulig et al^[39]^ found that Bcl-2 had lower expression in PCs than in all types of PAs and the normal pituitary, and this low expression corresponded to a high apoptotic index. Levels of Bax, Bcl-X, and Bad were also decreased in PCs compared with PAs. However, antiapoptotic Bcl-X_L_ was found in the majority of tissues, whereas pro-apoptotic Bcl-X_S_ was mostly absent or found at low levels, suggesting that Bcl-X proteins play a role in the apoptosis of pituitary neoplasms. In vitro, HP75 cells treated with TGF-β or PKC inhibitors demonstrated a 30% and 20% decrease, respectively, in Bcl-X levels compared to the control, whereas the expression of Bax and Bcl-2 was unchanged.^[50]^ These results reveal that PAs and PCs express both pro- and antiapoptotic proteins, which at first may appear contradictory. However, Soini et al^[51]^ have also reported a higher apoptotic index in malignant tumors, which is consistent with the findings of Kulig et al.^[39]^ In many cancers, apoptosis and proliferation are linked, although the underlying mechanisms remain unknown.^[51]^ Therefore, it is currently unclear whether Bcl-2 proteins can be used as biological markers of malignant transformation in PCs, and the correlation between proliferation and apoptosis in pituitary neoplasms should be further investigated.

#### 3.5.4 Metallothionein isoform 3

Metallothioneins (MTs) are a family of cysteine-rich short peptides comprising MT1, MT2, MT3, and MT4 isoforms that can bind both physiological and xenobiotic heavy metals. Functions of MT include heavy metal detoxification, scavenging of free radicals, and resistance to radiation; they are also involved in cell proliferation, differentiation, and apoptosis.^[52]^ Recent studies showed that deregulation of MT expression plays a role in different cancers.^[53,54]^

The MT3 isoform is brain specific and has an important function in nerve physiology and neuromodulation.^[55]^ MT3 may also play a role in PC development, as evidenced by its differential expression depending on tumor type. Thus, MT3 was found to be upregulated in ATCH-secreting and nonfunctioning PAs and downregulated in GH- and PRL-PAs compared with the normal pituitary; however, the correlation with hormonal secretion was not statistically significant, and the lowest MT3 level was observed in PCs.^[56]^ It has been suggested that MT downregulation in cancer is related to promoter hypermethylation as previously shown in hepatoma.^[57]^ It can thus be hypothesized that hypermethylation of the MT promoter may also occur in PCs, leading to a decrease in MT-mediated removal of free radicals, which may cause DNA damage and trigger cancer initiation. However, it has also been shown that MTs contribute to drug resistance in cancers.^[58]^ Overall, our systematic analysis indicates that the role of MTs in PC has been addressed in few studies, and comprehensive investigation is required to elucidate the biological relevance of MTs to PC formation and progression.

## 4 Discussion

### 4.1 Summary of the main findings

Our analysis identified several important genes and corresponding proteins involved in malignant transformation, growth, recurrence, metastasis, angiogenesis, and apoptosis in pituitary neoplasms (Table 2). Upregulation of cyclin D1, VEGF, MMP-9, miRNAs, and p21^Cip1^ showed a clear correlation with tumor cell cycle progression, vasculogenesis, metastasis, and invasion, and may contribute to malignant transformation of PAs to PCs. Downregulated factors such as MGMT, p16^Ink4A^, p27^Kip1^, Bcl-2, Bax, Bcl-x, and MT3 are involved in the cell cycle, apoptosis, tumor suppression, metabolism, and enhanced TMZ sensitivity. Figure 2 presents a schematic diagram showing the functions of the selected genes and proteins in PCs. These pathways suggest that the development and progress of PCs results from a multistage process with multigene mutations, similar to the pathogenesis of other cancers. Further research should more extensively evaluate the identified genes to provide a further understanding of the malignant transformation in PCs, and to validate these genes as candidate diagnostic markers and therapeutic targets in pituitary neoplasms.

**Figure:**
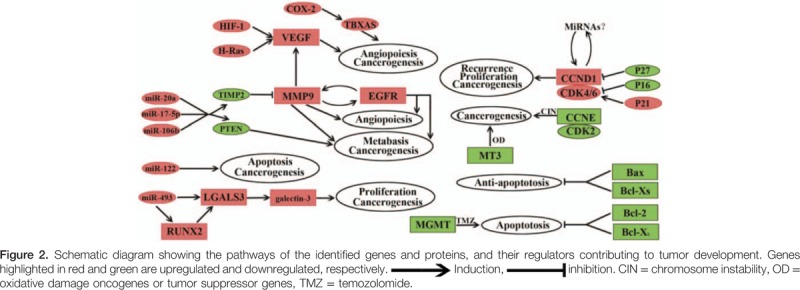


### 4.2 Study limitations

Despite strict adherence to the PRISMA guidelines, our systematic review has some limitations. The first limitation is related to the analytical methods used in the selected studies to analyze gene expression (Table 1). Of 32 studies, 27 used IHC as the main technique to detect protein expression, and only 5 of them used western blotting to validate the results; in addition, the changes in protein expression were not always confirmed at the mRNA level. Therefore, in these studies, the regulatory network of gene-to-protein expression was not comprehensively analyzed. However, we did not only focus on studies showing differential gene expression in PCs both at the mRNA and protein levels; we also included studies that analyzed post-transcriptional mechanisms for some genes such as *CDKN1B* (p27^Kip1^). These studies elucidated the regulatory cascade involved in the development of PCs and confirmed the role of the identified factors. Another limitation of our study is inherent to the rarity of PCs, which inevitably limits the sample size of the analyzed PC tumors. Thus, in most studies, the number of PC specimens was under 10 (data not shown), indicating weak statistical power and low statistical significance of the obtained results. In addition, PCs and PAs have variable endocrine activity, which is an important factor affecting gene expression; this issue additionally complicates the analysis because the segregation of tumors according to the type of hormonal secretion further decreases sample size. Few studies thus analyzed gene expression in pituitary tumors according to secretion type, and we did not separately discuss their findings.

### 4.3 Future research

The establishment of effective diagnostic and treatment targets for PCs is a challenge because of the rarity of the disease. This systematic review presents studies that obtained conflicting results regarding genetic changes in PC, complicating the comprehensive analysis of the involved genetic mechanisms. The differences in findings among studies may be attributed to flaws in experimental design. Therefore, in-depth analysis performed using advanced experimental methods should provide more reliable results. Cancer is a multistage process involving a number of mutated genes; therefore, molecular nodes in cancer signaling networks should be identified as potential precise therapeutic markers to be targeted simultaneously in PC treatment. This approach has proven effective in PC as evidenced by recent reports of cases treated with TMZ and CCNU in combination with 5-FU and bevacizumab, which target different molecular pathways involved in tumor development, emphasizing the need for further extensive genetic and epigenetic research regarding PC molecular biomarkers. This information will aid in early diagnosis and precise treatment of PCs, and eventually improve patients’ prognosis.

## 5 Conclusions

This review of comparative gene expression in PC revealed a number of potential candidate genes that may contribute to malignant transformation, angiogenesis, invasion, metastasis, and recurrence of PC. To our knowledge, this is the first systematic review that comprehensively analyzes genetic studies of PCs. Most of the identified genes and proteins have not been extensively evaluated with regard to the underlying mechanisms and diagnostic and therapeutic potential; however, they may be candidate targets for precise targeted gene therapies in the personalized treatment of PCs. Many of the described genes also play a crucial role in other human cancers, and some of them have already been used as effective treatment targets, underscoring the necessity of their evaluation for pituitary neoplasms, which would further the understanding of the transformation from benign pituitary tumors to malignant PCs. Considering the current shift of the treatment paradigm to personalized medicine, the identification of novel molecular biomarkers and targets might increase the precision and efficacy of therapies for PC, and such efforts will ultimately contribute to improving patients’ prognosis.
